# Additional Complexity in Historic and Contemporary Gene Flow Among Hoary, Vancouver Island, and Olympic Marmots Revealed by Microsatellites and Ultraconserved Elements

**DOI:** 10.1002/ece3.71711

**Published:** 2025-07-27

**Authors:** Natalie M. Hamilton, Nicholas J. Kerhoulas, Kathryn M. Everson, Aren M. Gunderson, Link E. Olson

**Affiliations:** ^1^ Department of Mammalogy University of Alaska Museum Fairbanks Alaska USA; ^2^ Department of Wildlife California State Polytechnic University, Humboldt Arcata California USA; ^3^ Department of Integrative Biology Oregon State University Corvallis Oregon USA

**Keywords:** introgression, *Marmota*, microsatellites, ultraconserved elements

## Abstract

Alpine species are inordinately threatened by habitat loss and precipitation changes resulting from climate change. In North America's Pacific Northwest (PNW), three closely related alpine mammal species—hoary, Olympic, and Vancouver Island marmots—may face greater negative impacts of climate change relative to species found at lower elevations. Phylogenetic studies have found these three species form a monophyletic complex; however, discordant evolutionary histories between mitochondrial and nuclear genes suggest that gene flow may have occurred between these marmot species. Furthermore, mitochondrial data find two reciprocally monophyletic mitochondrial clades (haploclades) of hoary marmots. Nuclear data do not recover this pattern, and interspecific relationships among the markers are not consistent. We used nine microsatellite loci and ultraconserved elements (UCEs) to explore patterns of nuclear gene flow among marmot species in the PNW. Analyses of microsatellite data indicate no current gene flow between hoary and Vancouver Island marmots or between hoary and Olympic marmots but do reveal nuclear gene flow among hoary marmot haploclades. Additionally, UCE data reveal historic gene flow between hoary and Vancouver Island marmots. Overall, our results suggest that historic mitochondrial introgression between hoary and Vancouver Island marmots, as well as male‐biased dispersal, are driving mito‐nuclear discordance in this species complex.

## Graphical abstract

This study examines gene flow among three alpine marmot species in the Pacific Northwest, focusing on the hoary, Olympic, and Vancouver Island marmots, which are highly threatened by climate change. Using microsatellite loci and ultraconserved elements, the research finds no current gene flow between hoary and Vancouver Island marmots but reveals complex patterns of historical and ongoing nuclear gene flow among hoary marmot populations. The findings suggest mitochondrial introgression and male‐biased dispersal as key factors contributing to genetic discordance, highlighting the need for further investigation to understand how these patterns may shift with climate change.

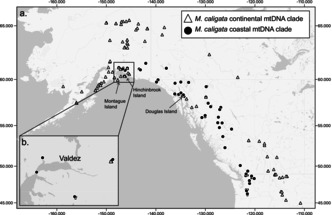

## 1 Introduction

Terrestrial vertebrate populations are experiencing extremely high rates of extirpation (Ceballos et al. 2017). This is likely the result of multiple factors, including climate and land‐use change (Masson‐Delmotte et al. 2021), which may disproportionately impact alpine species (Dirnböck et al. 2011; Rowe et al. 2015) as rates of warming, as well as the frequency and intensity of precipitation, are more pronounced at higher elevations (e.g., Pepin et al. 2015; Siirila‐Woodburn et al. 2021). While some populations may persist due to standing genetic variation or adaptive capacity, the survival of many populations will depend on their ability to either disperse among increasingly isolated habitat refugia or shift to different habitats entirely (Thurman et al. 2020). Because of their distribution on or near mountaintops, alpine‐dependent species may have limited potential to adapt to climate change through upslope migration (e.g., Stewart et al. 2015, 2017), which can also lead to population isolation and a commensurate reduction in both gene flow and genetic diversity (Wasserman et al. 2012). Lowered genetic diversity, in turn, can have short‐ and long‐term effects on population fitness and persistence. Decreased heterozygosity may increase the incidence of expression of deleterious alleles through inbreeding depression or genetic drift (Vranckx et al. 2012), ultimately leading to a reduction in adaptive potential and increased risk of extinction (Shirk and Cushman 2014). Although the significance of habitat connectivity for maintaining population viability is axiomatic, the specific factors that impact genetic structure and population connectivity remain largely unknown for many species. Understanding drivers of genetic structure can aid in population management of climate‐sensitive species, as population history can have a critical impact on adaptive evolutionary potential (e.g., Lanier et al. 2015). Characterization of genetic diversity, population structure, and phylogenetic patterns within and between species is therefore a critical first step in understanding how alpine‐restricted species may be affected by rapidly accelerating climate change.

**FIGURE 1:**
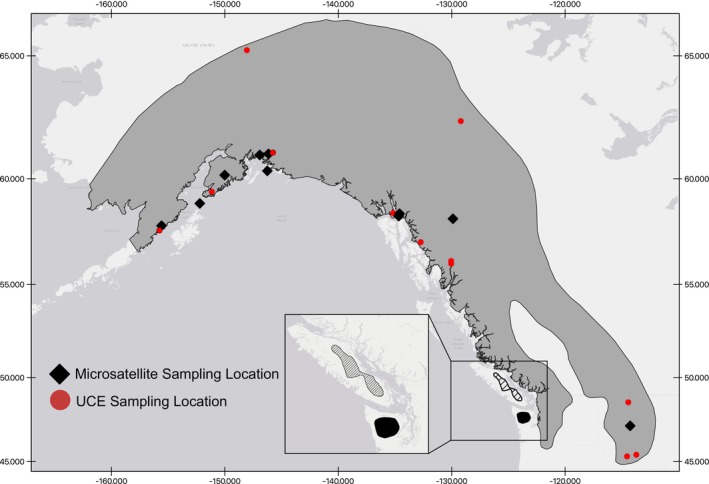
Distribution of Pacific Northwest marmot species and microsatellite sampling locations. The large gray region represents the distribution of *M. caligata* . The distributions of *M. olympus* (black region on the Olympic Peninsula) and *M. vancouverensis* (striped region on Vancouver Island) are shown in the inset (modified from Kerhoulas et al. 2015). Locations of *M. caligata* sampling are indicated with black diamonds (microsatellites) and red circles (ultraconserved elements). Specific sampling locations for *M. olympus* and *M. vancouverensis* are not marked on this figure.

In North America's Pacific Northwest (PNW), several species of marmot are dependent on or largely associated with alpine habitats and are likely already experiencing declines as a result of climate change (e.g., Thelin et al. 2018; Murphy‐Williams 2020). The hoary marmot, *Marmota caligata* (Eschscholtz, 1829), is the most broadly distributed of these species, ranging from southern Washington, central Idaho, and southern Montana north into Alaska and neighboring Yukon and Northwest Territories of Canada (Figure 1; Kerhoulas et al. 2015). The southernmost populations may harbor the highest levels of genetic diversity (Kerhoulas et al. 2015), but as these populations already occur near mountaintops, they have limited potential to move up in elevation to mitigate the effects of changing climate conditions. Moreover, unlike montane ecosystems throughout much of the rest of the *M. caligata* range, Washington's Cascade Mountains are largely discontinuous from one another, and the alpine‐subalpine ecotone occurs at higher elevations; collectively, this has likely resulted in reduced gene flow between isolated populations occurring on different mountains (Griffin 2007). The Vancouver Island marmot, *M. vancouverensis* Swarth, 1911, is endemic to Vancouver Island, British Columbia, Canada, and is classified as Critically Endangered by the International Union for Conservation of Nature (Roach 2017). Finally, the Olympic marmot, *M. olympus* Merriam, 1898, occurs only on Washington's Olympic Peninsula. This state‐endemic species has been a candidate for listing as Endangered, Threatened, or Sensitive at the state level since 2008 (Washington Department of Fish and Wildlife 2013, 2017, 2023) but currently lacks any formal state or federal conservation status. More recently, the Center for Biological Diversity has petitioned the US Fish and Wildlife Service to list the species as Threatened or Endangered under the federal Endangered Species Act (Kunkler 2024).

Previous research supports *M. caligata* , *M. olympus* , and *M. vancouverensis* as a monophyletic assemblage (Steppan et al. 2011; Kerhoulas et al. 2015; Mills et al. 2023). There is further evidence that isolation in separate refugia during the Last Glacial Maximum resulted in two reciprocally monophyletic mitochondrial (mtDNA) lineages of *M. caligata* (Kerhoulas et al. 2015; Lanier et al. 2015; Rankin et al. 2019; Mills et al. 2023): a coastal clade in the Cascade and Coastal mountains of the PNW from southern Washington north to Valdez, Alaska, and a continental clade ranging from the Rocky Mountains in southwestern Montana and central Idaho north to Interior, Southwestern, and Southcentral Alaska. These haplotype clades are known to be syntopic in the Interior Mountains of northern British Columbia near Dease Lake, as well as in the vicinity of Valdez, Alaska. However, nuclear‐based phylogenies do not recover the same two clades of *M. caligata* as mtDNA analyses (Kerhoulas et al. 2015; Mills et al. 2023). While nuclear genetic admixture is suggested between the coastal and continental mtDNA clades, patterns of gene flow and directionality remain unclear, as do taxonomic boundaries within the species as currently recognized (see below).

Additionally, the phylogenetic relationship between *M. caligata* and *M. vancouverensis* is unresolved. Analyses of mtDNA recover *M. vancouverensis* as sister to the coastal *M. caligata* haploclade. Despite the sister relationship between the *M. caligata* coastal haploclade and *M. vancouverensis* , extensive evidence—including nuclear and morphological data—supports *M. vancouverensis* as a distinct species (Cardini et al. 2009; Nagorsen and Cardini 2009; Kerhoulas et al. 2015; Mills et al. 2023). This mito‐nuclear discordance may be a result of incomplete lineage sorting and/or more recent interspecific gene flow between *M. caligata* and *M. vancouverensis* compared to a prior speciation event (Kerhoulas et al. 2015; Mills et al. 2023). Because *M. vancouverensis* has been the subject of substantial conservation efforts that include an ongoing captive breeding and release program, understanding the history of gene flow between these species may be critical to conservation, particularly if genetic rescue of *M. vancouverensis* becomes necessary (Hedrick and Fredrickson 2009).

The phylogenetic position of *M. olympus* relative to *M. caligata* and *M. vancouverensis* is likewise unresolved. Mitochondrial data recover *M. olympus* as sister to a clade composed of *M. caligata* haplogroups and *M. vancouverensis* (Steppan et al. 2011; Kerhoulas et al. 2015). In contrast, several nuclear alleles are shared exclusively between *M. olympus* and *M. caligata* from nearby populations in Washington (Kerhoulas et al. 2015). It remains unclear if these shared alleles are the result of incomplete lineage sorting, mitochondrial capture, historic and modern gene flow, or any combination thereof. Indeed, based on analysis of ultraconserved element (UCE) data, Mills et al. (2023) concluded that hoary marmots in the Cascade Mountains of Washington may represent a cryptic species and that *M. caligata* sensu lato may not be monophyletic. Given the restricted distribution of *M. olympus* , determining the extent of gene flow with *M. caligata* is beneficial for future conservation planning if genetic rescue of *M. olympus* becomes essential for its persistence.

In this study, we applied population genetic tools to analyze patterns of gene flow among *M. caligata* (see below), *M. olympus* , and *M. vancouverensis* . The goals of this study were to (1) clarify the distribution of the two *M. caligata* mtDNA clades and determine patterns of gene flow among them and (2) validate previous findings suggesting that *M. vancouverensis* captured the mitochondrial genome of coastal *M. caligata* . We exclude *M. caligata* specimens from Washington as investigations into cryptic speciation require taxonomic and geographic sampling beyond the scope of the current study.

## 2 Materials and Methods

### 2.1 DNA Extraction and Microsatellite Amplification

We amplified 15 microsatellite loci previously developed for use in marmots and closely related species (Table S1). Samples of *M. caligata* were included from 10 sampling locations across the hoary marmot's range, encompassing the latitudinal extent of both continental and coastal mtDNA clades (Alaska Peninsula *n* = 5, Douglas Island *n* = 11, Dease Lake British Columbia *n* = 3, Hinchinbrook Island *n* = 3, Juneau *n* = 8, Kenai Peninsula *n* = 23, Montana *n* = 4, Prince William Sound Continental mtDNA lineage *n* = 8, and the Prince William Sound Coastal mtDNA lineage *n* = 10). We also included samples of *M. olympus* (*n* = 5) and *M. vancouverensis* (*n* = 5). Amplification of microsatellite loci followed Schuelke (2000). For fragment analyses, reactions were combined by using 1.5 μL from each of three PCR reactions of independent loci (with different fluorescent dyes) for the same individual (Table S1) and brought to a final volume of 10 μL with 5.5 μL Hi‐Di Formamide (Applied Biosystems, Foster City, CA). Microsatellite loci were analyzed at the DNA Facility on Science Hill at Yale University using a Liz‐500 size standard (Applied Biosystems). Alleles were scored using GeneMarker v.2.6.0 (SoftGenetics, State College, PA).

To further delimit the distribution of hoary marmot mtDNA clades, we also extracted DNA from dried study skins or adventitious tissue removed from skeletal material from 138 museum specimens for which archived fresh tissues were not available (Table S2). DNA extractions were conducted in the University of Alaska Museum's ancient DNA extraction facility using the Promega DNA IQ system (Promega Corp., Madison, WI) following the manufacturer's protocol. We used one general mammalian primer and one specifically designed for *M. caligata* to amplify and sequence overlapping fragments of the mitochondrial cytochrome‐*b* gene (Table S1). PCR thermal‐cycling parameters followed Kerhoulas and Arbogast (2010) but used an annealing temperature of 48°C for 1.5 min (see Supporting Information for additional details). We then determined clade assignments via Sanger sequencing and/or the use of a restriction enzyme. In the latter case, we added 3.75 μL of NE Buffer 3 and 0.35 μL of restriction enzyme Bs1I (New England BioLabs, Ipswich, MA) to 25 μL of each successful PCR reaction and incubated at 55°C for 70 min, followed by 80°C for 20 min, and finally held at 4°C. Selection of this restriction enzyme was based on sequence data from 167 *M. caligata* specimens with known clade membership (Kerhoulas et al. 2015). Gel electrophoresis was used to visualize the size and number of fragments for clade assignment. Members of the coastal haplotype clade lacked a binding site for this restriction enzyme and produced a single (222 bp) band, whereas members of the continental haplotype clade had a single binding site and produced bands at 91 and 131 bp.

### 2.2 Analysis of Genetic Diversity and Population Structure

Microsatellite loci passing initial screening (see Section 3) were tested for deviation from Hardy–Weinberg equilibrium at each sampling location and across all locations combined using SPAGeDI v.1.5 (Hardy and Vekemans 2002) with a 1,000,000 Markov chain including 100,000 dememorization steps. We determined the observed and expected heterozygosity and unique alleles in SPAGeDi. Linkage between markers was investigated using ARLEQUIN v.3.5.2.2 (Excoffier et al. 2005), with 10,000 permutations and significance corrected for multiple comparisons (0.05/36 = 0.001). Pairwise *F*_ST_ values were calculated for each pair of *M. caligata* sampling sites using ARLEQUIN. Significance was assessed by *p* values calculated using 10,000 permutations and a Bonferroni correction for multiple comparisons (Rice 1989). To more directly test if molecular diversity in *M. caligata* is best partitioned by separating populations based on mitochondrial clades, we performed an analysis of molecular variance (AMOVA) using microsatellite data in ARLEQUIN with 10,000 permutations. Individuals were assigned to the coastal or continental group based on mitochondrial haplotype identity. Finally, to test for associations between genetic population differentiation (Slatkin linearized value; *F*_ST_/[1 − *F*_ST_]) and geographic distance (log transformation of shortest distances between colonies, ignoring elevational changes), we ran a Mantel test using the R‐package “vegan” with 10,000 permutations (Dixon 2003). Distance between colonies was computed in GenAlEx v.6.5 (Peakall and Smouse 2012).

We assessed population structure by first analyzing microsatellite data for *M. caligata* , *M. olympus* , and *M. vancouverensis* in STRUCTURE v.2.3.4 (Falush et al. 2007). We used the sampling location for each individual as prior information using the LOCPRIOR model (Hubisz et al. 2009). Model parameters were set to admixture with correlated allele frequencies, and we performed 10 replicate runs for each *K* value (1–11; the number of sampled populations), with a burn‐in of 1 × 10^4^ and 1 × 10^5^ MCMC repetitions. To identify the most appropriate *K* from our data, we viewed results in Structure Selector (Li and Liu 2018) and the most likely *K* was chosen from the highest log‐likelihood value. We also used the *adegenet* package (Jombart 2008) in *R* to perform multivariate analysis of population structure. First, geographic sampling location (or species in the case of *M. olympus* and *M. vancouverensis* ) was used as predefined groups for discriminant analysis of principal components (DAPC; Jombart et al. 2010). We used the *optim.a.score* function to determine the optimal number of principal components to retain to best describe the population structure while avoiding overfitting the discriminant functions. Then, we performed this same DAPC workflow using populations that were defined by probable clusters found using the *find.clusters* algorithm.

To explore contemporary migration rates, we analyzed microsatellite data using the program BA3 v.3.0.5.6 (Wilson and Rannala 2003). This software uses a Bayesian inference approach to estimate recent migration rates between populations within the last several generations (*m*). We tested three different population groupings: individuals assigned to populations based on (1) sampling location, (2) species, and (3) mitochondrial lineages (coastal and continental). Then, we conducted preliminary runs and adjusted mixing parameters to ensure acceptance rates were as close to the 20%–60% recommended range as possible. Finally, we conducted five full runs (‐*i* = 10,000,000, ‐*b* = 1,000,000, and ‐*n* = 1000) using different starting seeds. Convergence of parameter estimates was assessed by examination of trace files using Tracer v.1.7.2 (Rambaut et al. 2018) and the best run was determined by Bayesian deviance using the *calculateDeviance.R* script (Meirmans 2014).

### 2.3 Investigating Causes of Mitochondrial‐Nuclear Discordance and Introgression

We leveraged genomic data from Mills et al. (2023) to examine the hypothesis that mitochondrial introgression explains the apparent sister relationship between *M. vancouverensis* and the coastal mtDNA clade of *M. caligata* (Table S3) in phylogenetic analyses of mtDNA. UCE contigs were downloaded for *M. caligata* (*n* = 15; continental mtDNA clade = 9, coastal mtDNA clade = 6), *M. olympus* (*n* = 4), *M. vancouverensis* (*n* = 2), and *M. flaviventris* (*n* = 1) as an outgroup (Mills et al. 2023; Table S3). UCE data were processed using Phyluce 1.7.3 (Faircloth 2016). UCE matches were extracted from contigs using the *phyluce_assembly_match_contigs_to_probes*, and each locus was aligned using MAFFT v. 7.475. Then, we removed any loci that had data from fewer than 75% of the individuals (*phyluce_align_get_only_loci_with_min_taxa*). Raw data were also provided for these sequences, and we called SNPs using the workflow published in Erickson et al. (2021) (https://github.com/NmHamilton/Marmot_Historic_Contemporary_GeneFlow). These SNPs were filtered to keep only biallelic SNPs, and sites were thinned so that no two sites were within 1000 bp of one another (roughly one SNP per UCE locus to exclude linked loci), resulting in a final dataset that included 2963 SNPs. More information for UCE samples can be found in Figure 1 and Table S3.

To assess the potential for introgression to contribute to topological variation between *M. vancouverensis* and *M. caligata* coastal and continental mitochondrial clades, we used the *f*‐branch statistic in *Dsuite v.0.4* (Malinsky et al. 2020) for biallelic SNPs called from UCEs. We first used the “DtriosCombine” function to calculate genome‐wide f4‐ratio statistics for all possible trios of species using the nuDNA tree as a starting topology. This tree was obtained from pruning the concatenated UCE tree found in Mills et al. (2023). Samples were assigned to one of four groups: *M. caligata* coastal, *M. caligata* continental, *M. olympus* , and *M. vancouverensis* . *Marmota caligata* specimens were assigned to coastal and continental clades based on results of Mills et al. (2023) or Kerhoulas et al. (2015). The *f*‐branch statistics were visualized using the “dtools.py” utility included within *Dsuite*.

## 3 Results

**FIGURE 2:**
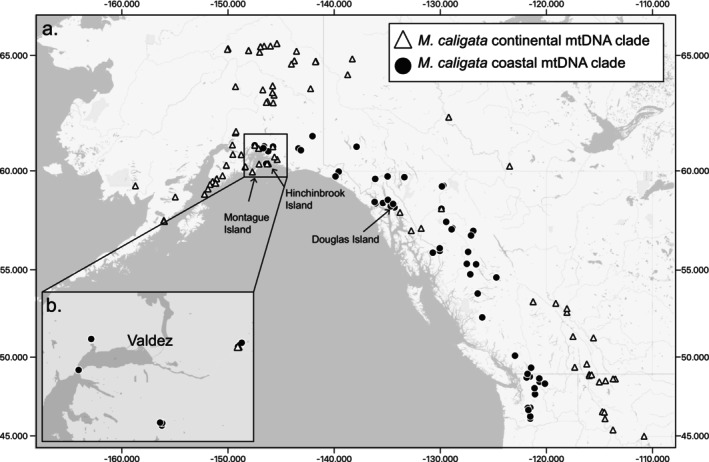
(a) Distribution of *M. caligata* specimens and mitochondrial (mtDNA) clade membership. Only specimens with a coordinate uncertainty ≤ 25 km are shown. (b) Detailed view of sampling in Valdez and surrounding area.

We determined the mtDNA haplotype clade membership for 98 out of 138 sampled *M. caligata* museum specimens that lacked archived fresh tissue (Figure 2; Table S2). Of the museum specimens assigned to a particular mtDNA clade, Sanger sequencing and restriction enzymes were used to classify 17 and 81 specimens, respectively. For all but two of these (USMN 241748 and MVZ 964), the geographic distribution of clades was comparable to the distribution of 167 specimens previously assigned in Kerhoulas et al. (2015). USNM 241748 was assigned to the coastal clade but is from Interior Alaska, a region otherwise occupied exclusively by the continental clade, and thus likely represents contamination. Specimen MVZ 964 was collected on Hinchinbrook Island, AK, where only continental‐clade specimens have been previously documented (Kerhoulas et al. 2015). The assignment of this specimen to the coastal clade is ambiguous for a number of reasons. Coastal clade specimens have been collected from the mainland within 65 km of Hinchinbrook Island, but previous molecular analyses of specimens with archived fresh tissue placed Hinchinbrook Island *M. caligata* in the continental clade (Kerhoulas et al. 2015). The specimen in question was collected from a different region of the island more than 100 years before those assigned to the continental clade were collected; the island therefore could be home to both haplotype clades and represent multiple colonization events. We ultimately excluded these two questionable specimens from further analyses and confidently assigned 46 and 50 specimens to the coastal and continental clades, respectively.

### 3.1 Population Genetics

Nine of the 15 microsatellite loci screened produced usable results. The six omitted loci (2g2, MA066, MA001, MA091, ST10, and SS‐Bibl25) had regions of ambiguous peaks and/or produced a product greatly different in size than previously observed in marmots or closely related species. We found no deficiency in heterozygotes or significant linkage disequilibrium among loci using a Bonferroni‐corrected alpha value that accounted for multiple tests (i.e., *α* = 0.05/8 = 0.00625; Table S5). Among *M. caligata* populations, pairwise *F*_ST_ ranged from 0.016 (between the coastal and continental Prince William Sound populations) to 0.854 (between Hinchinbrook Island and the Alaska Peninsula; Table 1). For *M. olympus* and *M. vancouverensis* , pairwise *F*_ST_ values among all other localities were generally high (range: 0.487–0.873). AMOVA results (Table 2) indicated that the majority of genetic variance was found within geographic sampling locations (76.32%, *p* < 0.001), followed by among populations (22.89%, *p* < 0.001). Genetic variance was not found to be significantly different among the coastal and continental mitochondrial *M. caligata* clades (0.78%, *p* = 0.310). The Mantel test found no significant isolation by distance (IBD) among *M. caligata* sample sites (*r* = 0.0776, *p* = 0.332).

**TABLE 1:** Pairwise *F*_ST_ among all populations using microsatellite data. Significant values are in bold.

	Alaska Peninsula	Douglas Island	Dease Lake	Hinchinbrook Island	Juneau	Kenai Peninsula	Montana	Prince William Sound Coastal	Prince William Sound Continental	*M. olympus*	*M. vancouverensis*
Alaska Peninsula	0										
Douglas Island	**0.525**	0									
Dease Lake	**0.541**	0.37	0								
Hinchinbrook Island	0.854	0.546	0.544	0							
Juneau	**0.386**	**0.129**	0.244	0.403	0						
Kenai Peninsula	**0.279**	**0.229**	**0.277**	**0.331**	**0.083**	0					
Montana	0.436	0.339	0.21	0.479	0.229	**0.293**	0				
Prince William Sound Coastal	**0.337**	**0.221**	0.328	0.502	0.031	0.063	0.307	0			
Prince William Sound Continental	**0.334**	**0.231**	0.251	0.421	0.049	0.028	**0.295**	0.017	0		
*M. olympus*	0.766	**0.648**	0.538	0.811	0.566	**0.565**	0.487	**0.613**	**0.581**	0	
*M. vancouverensis*	0.814	**0.673**	0.585	0.873	0.598	**0.598**	0.529	**0.598**	**0.626**	0.737	0

**TABLE 2:** Results of the analysis of molecular variance (AMOVA) using microsatellite data for *M. caligata* mtDNA clades. Individual *M. caligata* were placed into one of two groups based on mtDNA identity (coastal or continental).

Coastal and continental mtDNA *M. caligata* clades, Number of groups = 2						
Source of variation	df	Sum of squares	Variance components	Percentage of variation	Fixation index	*p*
Among groups	1	16.729	0.020	0.78	0.0078 (*F*_CT_)	0.31
Among populations within groups	7	74.478	0.592	22.89	0.2307 (*F*_SC_)	< 0.0001
Within populations	141	278.153	1.973	76.32	0.2554 (*F*_ST_)	< 0.0001
Total	149	369.360	2.585			

**FIGURE 3:**
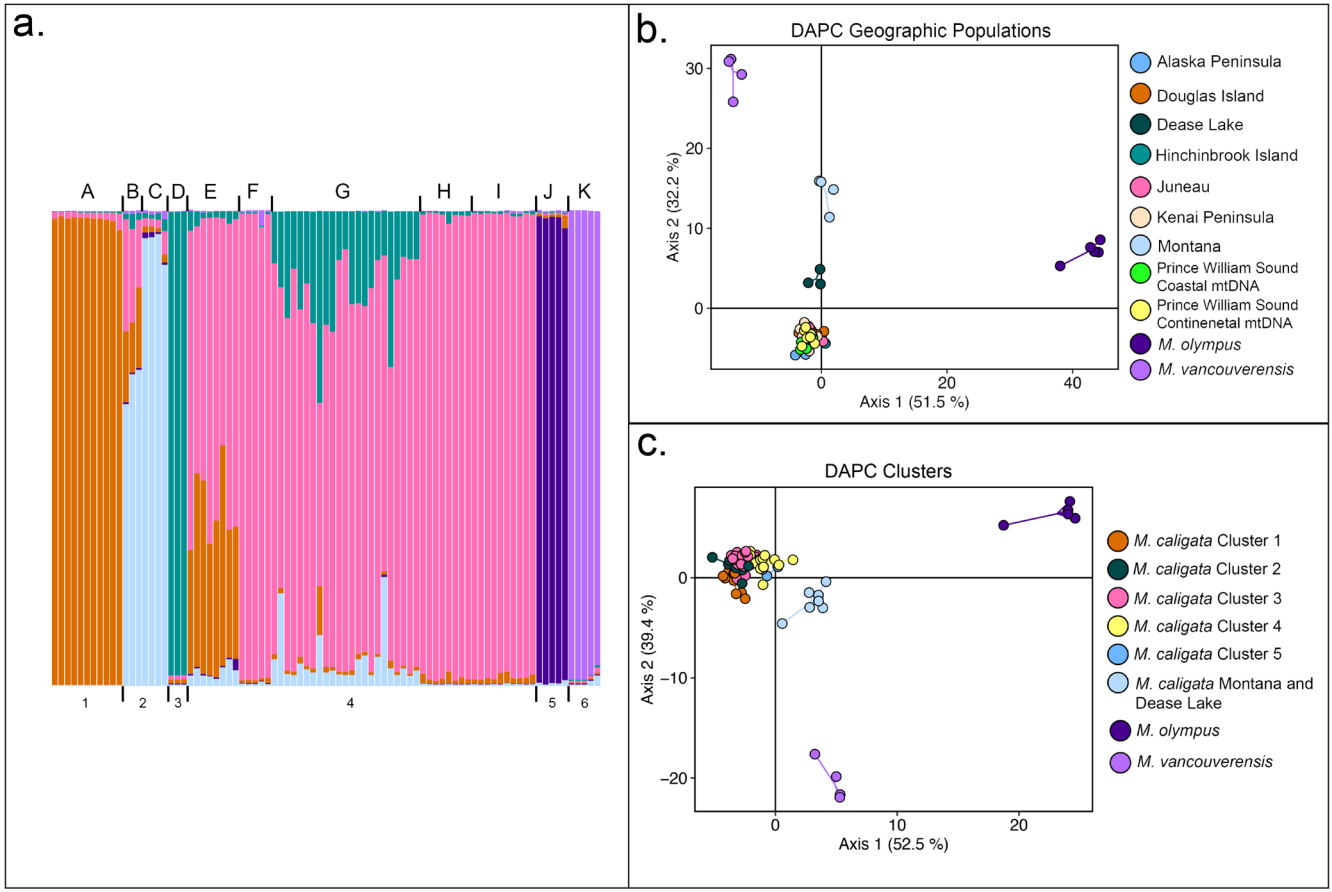
Results of population structure analyses of PNW *Marmota* using microsatellite genotype data. (a) Admixture plot of results of STRUCTURE analysis (*K* = 6) of nine microsatellite loci for *M. caligata* , *M. olympus* , and *M. vancouverensis* . Each bar represents an individual and the color indicates the proportion of each *K* population the individual is assigned to. Letters above bars indicate geographic sampling location: (A) Douglas Island, (B) Dease Lake, (C) Montana, (D) Hinchinbrook Island, (E) Juneau and the surrounding area, (F) Alaska Peninsula, (G) Kenai Peninsula, (H) Prince William Sound (continental), (I) Prince William Sound (coastal), (J) *M. olympus* , and (K) *M. vancouverensis*. Numbers correspond to population assignment from STRUCTURE. (b) DAPC results from populations partitioned by geographic sampling location. Each node represents the genotype of an individual connected to a centroid of the cluster the individual was assigned to, colored by geographic sampling location. (c) DAPC results from populations partitioned by *find.clusters* algorithm where *K* = 8.

Analyses in STRUCTURE indicated *K* = 6 was the best‐fit model using the peak mean probability method (Figure 3a; Figure S1). Well‐defined clusters were recovered for *M. olympus* and *M. vancouverensis* as well as *M. caligata* from Douglas Island and Hinchinbrook Island. Specimens from Dease Lake and Montana formed a cluster and the remaining *M. caligata* from the Alaska Peninsula, Juneau, Kenai Peninsula, and both mtDNA clades from Prince William Sound (PWS) form the final group (Figure 3a). There is evidence of admixture among the Douglas Island, Dease Lake, and Juneau populations. There is also admixture among K4 (Juneau, Kenai Peninsula, and Prince William Sound populations), Alaska Peninsula, Dease Lake, and Hinchinbrook Island. DAPC analyses produced similar results. When we partitioned populations based on sampling location, the first two discriminant functions explained 51.5% and 32.2% of the genetic variation from sampled sites (Figure 3b). Visual inspection of the resulting plot shows significant overlap between *M. caligata* from the Kenai Peninsula, Prince William Sound (both mitochondrial clades), Alaska Peninsula, Douglas Island, Juneau, and Hinchinbrook Island (Figure 3b). *Marmota olympus* and *M. vancouverensis* each occupy distinct regions of the DAPC analysis. Specimens from Montana and Dease Lake formed distinct clusters. We found similar patterns when we partitioned populations based on the *find.clusters* function (*K* = 8), wherein individuals were not assigned to their original geographic sampling locations but rather into eight different groups, similar to those identified in STRUCTURE analyses. Using *find.clusters*, *M. caligata* from Montana and Dease Lake were assigned to a single group (Figure 3c). *Marmota olympus* and *M. vancouverensis* each formed unique groups. The remaining *M. caligata* specimens were recovered in five different groups, indicating some admixture between populations. Although the remaining *M. caligata* specimens were placed into different clusters, there is significant overlap similar to that seen in the first DAPC analysis. When populations were thus assigned, axis 1 explained 52.5% of the variation and axis 2 explained 39.4% of the variation.

### 3.2 Contemporary Migration Rates

The best population grouping for contemporary gene flow in BA3, determined by Bayesian deviance, was the model that split individuals by geographic sampling location (Table S5). Analyses indicated unidirectional gene flow from populations on the Kenai Peninsula to Alaska Peninsula (*m* = 0.1242 ± 0.0374), Douglas Island (0.1244 ± 0.0391), Juneau (0.1571 ± 0.0374), and the two Prince William Sound haploclades (Continental: 0.1566 ± 0.0372; Coastal: 0.1752 ± 0.0355). We found no evidence of gene flow between the Prince William Sound Continental and Coastal haploclades. The remaining migration 95% confidence intervals overlapped zero and were therefore nonsignificant.

### 3.3 Introgression

**FIGURE 4:**
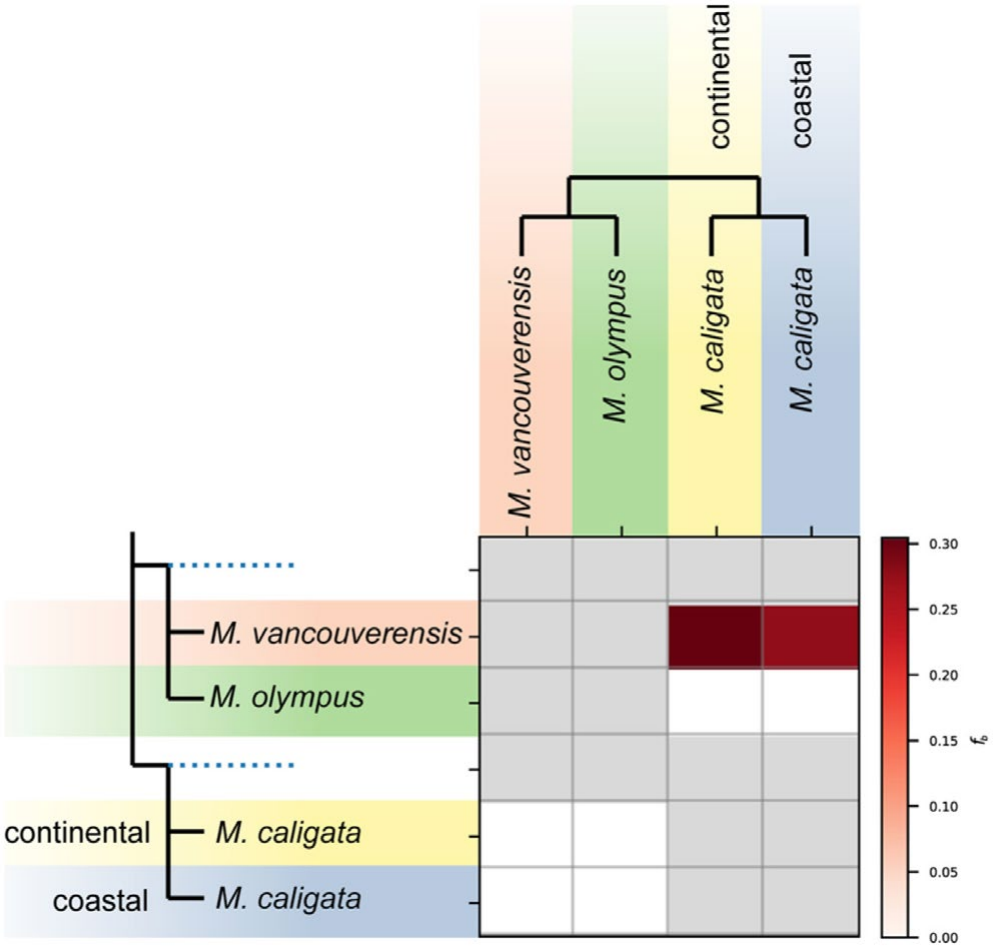
Matrix of *f*‐branch values from the Dsuite analysis of ultraconserved elements using the nuDNA tree topology. These values represent the proportion of alleles shared between the donor (column) and recipient (row) branches in excess of that predicted by the multispecies coalescent model, indicating likely instances of introgression.

Using UCE data from Mills et al. (2023), we investigated potential introgression between PNW marmot species using *D*‐statistics. We found strong signals of introgression between *M. vancouverensis* and both the *M. caligata* continental and *M. caligata* coastal haplotype clades (33% and 31% respectively; Figure 4).

## 4 Discussion

To better understand the complex history of gene flow and mechanisms underlying mito‐nuclear discordance in PNW marmot species, we subjected microsatellite and nuclear genomic data to a range of population genetics analyses. Our results suggest contemporary intraspecific gene flow among *M. caligata* mitochondrial clades. The inferred lack of contemporary gene flow between *M. caligata* and *M. vancouverensis* (Table S5), along with evidence of historic interspecific gene flow (Figure 4), indicates mito‐nuclear discordance is likely due to past introgression rather than ongoing gene flow between species.

### 4.1 Gene Flow

We examined inter‐ and intraspecific contemporary gene flow between PNW marmot lineages using nine microsatellite loci. Results of BA3, STRUCTURE, and DAPC analyses support gene flow between coastal and continental mtDNA clades of *M. caligata* as these analyses did not recover population clusters congruent with coastal and continental mtDNA clades (Figure 3). This discordance between mitochondrial and nuclear genetic structure, combined with the limited geographic overlap of the mtDNA clades, is consistent with sex‐biased gene flow. Specifically, the observed pattern may reflect male‐biased dispersal. Because mitochondrial DNA is maternally inherited, it reflects only the movement of females, whereas biparentally inherited nuclear markers capture gene flow from both sexes. In support of this explanation, male hoary marmots near Kluane National Park, Yukon, Canada have been shown to disperse from the natal colony at higher rates than females (Kyle et al. 2007), and male‐biased dispersal has also been documented in the closely related yellow‐bellied marmot (Downhower and Armitage 1981) and many other species of mammals (Greenwood 1980).

Distributional limits of the *M. caligata* mtDNA clades were clarified by sampling 98 historic museum specimens (Figure 2). *Marmota caligata* from Montague (from which there are no recent specimens) and Hinchinbrook Islands, Alaska, belong to the continental haploclade (Figure 2), suggesting that this haplotype clade was likely first to reach this region. Additionally, marmots from Douglas Island (coastal haploclade) and Hinchinbrook Island formed unique or nearly unique genetic clusters in the STRUCTURE analyses. Notably, the patterns of admixture found in nearby mainland populations (Juneau in the case of Douglas Island, and Prince William Sound for Hinchinbrook Island) suggest mainland populations as a source for these island populations. As both islands were likely overrun with ice during the LGM (Kaufman and Manley 2004) and thus colonized post‐LGM, the presence of distinct genetic clusters suggests both limited overwater dispersal in the species and that the markers used were sufficient to detect recent population isolation. This is particularly surprising in the case of the Douglas Island marmots given the very narrow water barrier between it and the mainland (The Gastineau Channel); in places, the extreme minus tide (−0.42 m) makes the channel crossable by walking.

The Interior Mountains of northern British Columbia connect the Coastal and Rocky Mountains, suggesting they would likely be the first place the two *M. caligata* mitochondrial clades would have experienced secondary contact, assuming post‐Pleistocene expansion from southern and/or coastal refugia (Kerhoulas et al. 2015). The prevalence of the coastal haploclade in this region (Figure 2) may suggest a nearby coastal refugium in northern British Columbia and/or Southeast Alaska facilitated early colonization. Members of both haplotype clades currently occur in this region; however, additional sampling from the northwestern portion of British Columbia may recover additional continental haplotypes. It is important to note that nearly half of the specimens sampled from northern British Columbia were collected over a century ago and thus may not be entirely representative of the current distribution of the haplotype clades. Despite the expansive distribution of *M. caligata* in western Canada, museum specimens with fresh tissue samples are extremely limited, with only 10 from British Columbia (representing 3 localities) and 2 from the Northwest Territories (from a single locality). In the other region of *M. caligata* haploclade sympatry near Valdez, Alaska, both clades were found in similar numbers in syntopy (Figure 2). In this region, specimens from both mtDNA clades were assigned to the same genetic cluster based on nuclear data (i.e., Prince William Sound continental and coastal populations; Figure 3b,c), suggesting ongoing gene flow between the haploclades, as predicted by our BA3 results (Table S5).

### 4.2 Interspecific Gene Flow and Introgression

Our results support previous findings that *M. vancouverensis* recently captured the mitochondrial genome of coastal *M. caligata* (Kerhoulas et al. 2015; Mills et al. 2023). *F*‐branch analyses suggest introgression between *M. vancouverensis* and both *M. caligata* haploclades. Notably, STRUCTURE and DAPC analyses of nuclear loci found *M. vancouverensis* forms a unique genetic cluster, with a small (< 5%) amount of admixture with *M. caligata* populations. BA3 did not support contemporary gene flow between *M. vancouverensis* and any *M. caligata* population. These results, combined with the morphological distinctiveness of *M. vancouverensis* (Cardini et al. 2007, 2009), suggest genetic exchange with *M. caligata* was limited, thereby preserving the majority of the nuclear diversity of *M. vancouverensis* . Hybridization is not uncommon in Marmotini, with up to 25% of species showing evidence of past or present gene flow, ranging from occasional hybridization events to broad zones of introgression (Brandler et al. 2021). In marmots, such hybridization events have been documented in *M. baibacina* (Kastschenko, 1899) where the range overlaps with *M. bobak* (P.L.S. Müller, 1776), *M. caudata* (L. Geoffroy Saint‐Hilaire, 1844), and *M. sibirica* (von Radde, 1862) (Brandler et al. 2021). While *M. caligata* and *M. vancouverensis* are not currently sympatric, there have been an increasing number of observations of *M. caligata* in mainland Vancouver near the Strait of Georgia (which separates Vancouver and Vancouver Island; iNaturalist). Given that marmots are capable of long‐distance hitchhiking in the undercarriage of cars (Eastman 2022; Kloster 2020), this may be cause for concern for the protected *M. vancouverensis* . Although our analyses do not find evidence of ongoing gene flow between species, they do reveal historic introgression, highlighting that hybridization has occurred under appropriate conditions in the past. As climatic barriers decrease and unintentional translocations of marmots by humans continue, there is an increased potential for secondary contact and subsequent hybridization, which has important implications for the conservation of *M. vancouverensis* .

Furthermore, STRUCTURE analyses resolved *M. olympus* as a distinct population, and BA3 results suggest no contemporary gene flow between *M. olympus* and any other populations we included (Figure 3; Table S5). However, it is important to note that *M. caligata* from Washington was excluded from this study due to previous findings of cryptic speciation in these populations (Kerhoulas et al. 2015; Mills et al. 2023). More extensive sampling (geographic and genomic) is necessary to illuminate the extent of historic and contemporary gene flow between *M. caligata* and *M. olympus* .

## 5 Conclusions

Our analyses of microsatellite and UCE loci support historic introgression of the coastal *M. caligata* mitochondrial genome into *M. vancouverensis* , likely during the Last Glacial Maximum. Despite this mitochondrial capture, *M. vancouverensis* maintains a distinct nuclear genetic identity with minimal evidence of recent admixture. The incongruence between nuclear and mitochondrial phylogenies, combined with the limited sympatry observed between mtDNA clades, suggest that male‐biased dispersal plays a key role in shaping gene flow within *M. caligata* . These results emphasize the importance of distinguishing between historical and contemporary gene flow when interpreting patterns of hybridization, particularly in the context of climate change. As species' ranges shift and phenologies change in response to warming climates, opportunities for secondary contact and hybridization are expected to increase (Vallejo‐Marín and Hiscock 2016). Therefore, future work on climate‐sensitive species should integrate multilocus data and temporally explicit analyses.

Additionally, within *M. caligata* , nuclear genetic data reveal substantial admixture across populations, with the exception of the most geographically isolated southern populations (Dease Lake and Montana), which form unique genetic clusters. Admixture among populations on the Alaska Peninsula, Kenai Peninsula, Prince William Sound, and Juneau suggests that *M. caligata* is able to disperse through non‐alpine habitat. This is supported by long‐standing sea‐level records across Southeast, Southcentral, and Western Alaska, including both historic observations (e.g., Heller 1910) and our more recent field surveys. The capacity of *M. caligata* to disperse through and occupy non‐alpine habitat adds to a growing body of literature that suggests some alpine mammals may be capable of long‐distance dispersal across areas of low elevation. For example, genetic studies have found evidence of unexpected dispersal potential in species such as the yellow‐bellied marmot and mountain goat (Floyd et al. 2005; Shafer et al. 2010). This is in contrast to the American ( *Ochotona princeps* (J. Richardson, 1828)) and collared pika ( *Ochotona collaris* (E.W. Nelson, 1893)), which largely exhibit greater population structure, particularly in fragmented and lower latitude portions of their range (Galbreath et al. 2009; Waterhouse et al. 2017; Klingler et al. 2021). However, research in collared pika has demonstrated that even limited gene flow can play a role in restoring critical genetic diversity in small or isolated alpine mammals (Zgurski et al. 2014). In this context, our findings of gene flow in *M. caligata* suggest this species may have greater ecological flexibility than previously expected, and may be buffered from the immediate genetic consequences of climate‐induced range shifts. Nevertheless, continued dispersal between mountain ranges may become increasingly difficult for all alpine mammals as climate change reduces the suitability of intervening lowland habitats (Floyd et al. 2005; Schloss et al. 2012).

Thus, future research incorporating both broader and fine‐scale geographic sampling is critical to better understand how landscape and climatic features influence gene flow in *M. caligata* . Specifically, targeted sampling of island populations would shed light on the frequency of overwater dispersal. Increased sampling in British Columbia and Washington is also essential for clarifying taxonomic boundaries of southern populations, which our current sampling was insufficient to adequately assess. Overall, this study provides important insights into the phylogeography of *M. caligata* and will hopefully guide much‐needed future work on the taxonomy, conservation, and biogeography of North America's marmots.

## Author Contributions

**Natalie M. Hamilton:** conceptualization (equal), formal analysis (lead), methodology (equal), writing – original draft (equal). **Nicholas J. Kerhoulas:** conceptualization (equal), data curation (equal), formal analysis (supporting), funding acquisition (equal), writing – original draft (supporting), writing – review and editing (equal). **Kathryn M. Everson:** data curation (equal), resources (equal), writing – review and editing (equal). **Aren M. Gunderson:** funding acquisition (equal), resources (equal), writing – review and editing (equal). **Link E. Olson:** conceptualization (equal), funding acquisition (equal), project administration (lead), resources (equal), supervision (lead), writing – review and editing (lead).

## Conflicts of Interest

The authors declare no conflicts of interest.

## Supporting information


**Table S1.** Sequence information for all microsatellite loci tested for *Marmota* species in this study. Information on fluorescent primers used with each locus are also included.

**Table S2.** *Marmota* specimens used in this study. Museum abbreviations are as follows: MCZ = Museum of Comparative Zoology, Harvard University, Cambridge, Massachusetts; MSB = Museum of Southwestern Biology, University of New Mexico, Albuquerque, New Mexico; MVZ = Museum of Vertebrate Zoology, University of California, Berkeley, California; ROM = Royal Ontario Museum, Toronto, Ontario; UAM = University of Alaska Museum, Fairbanks, Alaska; USNM = National Museum of Natural History, Smithsonian Institution, Washington D.C.; UWBM = University of Washington Burke Museum of Natural History and Culture, Seattle, Washington; YPM = Yale Peabody Museum of Natural History, Yale University, New Haven, Connecticut. Mitochondrial clade membership for *M. caligata* specimens previously determined using Sanger sequencing of fresh tissue are denoted by plain text. Clade assignments for museum specimens with no archived tissue samples were made using restriction enzymes (bold) or Sanger sequencing (bold italic). Specimens used in microsatellite analyses indicated with an asterisk after the species name and genotypes are reported (e.g., microsatellite loci 3b1 and GS25).

**Table S3.** Individuals from Mills et al. (2023) that were used for introgression analyses. Clade identity refers to whether the specimen falls into the coastal or continental mitochondrial lineage. Clade identity was determined either by Sanger sequencing for this project (see Table S2) or by mitochondrial phylogenomic analyses in Mills et al. (2023).

**Table S4.** Diversity measures for each *M. caligata* sampling location, *M. olympus* , and *M. vancouverensis* . *F*_IS_ = inbreeding coefficient, *H*_e_ = expected heterozygosity, *H*_o_ = observed heterozygosity, *p* = significance value for inbreeding.

**Table S5.** Results of the top BA3 model. The Source → Sink populations represent the direction of tested migration: from the source population to the sink population. Mean represents the proportion of individuals from the sink population that are from the source population. Migration patterns with confidence intervals that do not overlap zero are highlighted.

**Figure S1.** Results of STRUCTURE analysis. Mean estimated log‐normal (Ln) probability of the data in relation to the simulated number of clusters K. Vertical bars indicate standard deviation among ten replicates.

## Data Availability

Our complete analytical dataset is available on Dryad: https://doi.org/10.5061/dryad.tb2rbp0bc. Microsatellite data are provided as Table S2, and UCE data can be found on our Dryad repository. Scripts and walkthroughs can be found on GitHub: https://github.com/NmHamilton/Marmot_Historic_Contemporary_GeneFlow[dataset].
