# Supplementary figures and images for: Additional Complexity in Historic and Contemporary Gene Flow Among Hoary, Vancouver Island, and Olympic Marmots Revealed by Microsatellites and Ultraconserved Elements
Source: Ecol Evol. 2025 Jul 27;15(7):e71711. doi: 10.1002/ece3.71711 (PMC12301068; doi:10.1002/ece3.71711)

Mean LnP(K)  $\pm$  Stdev

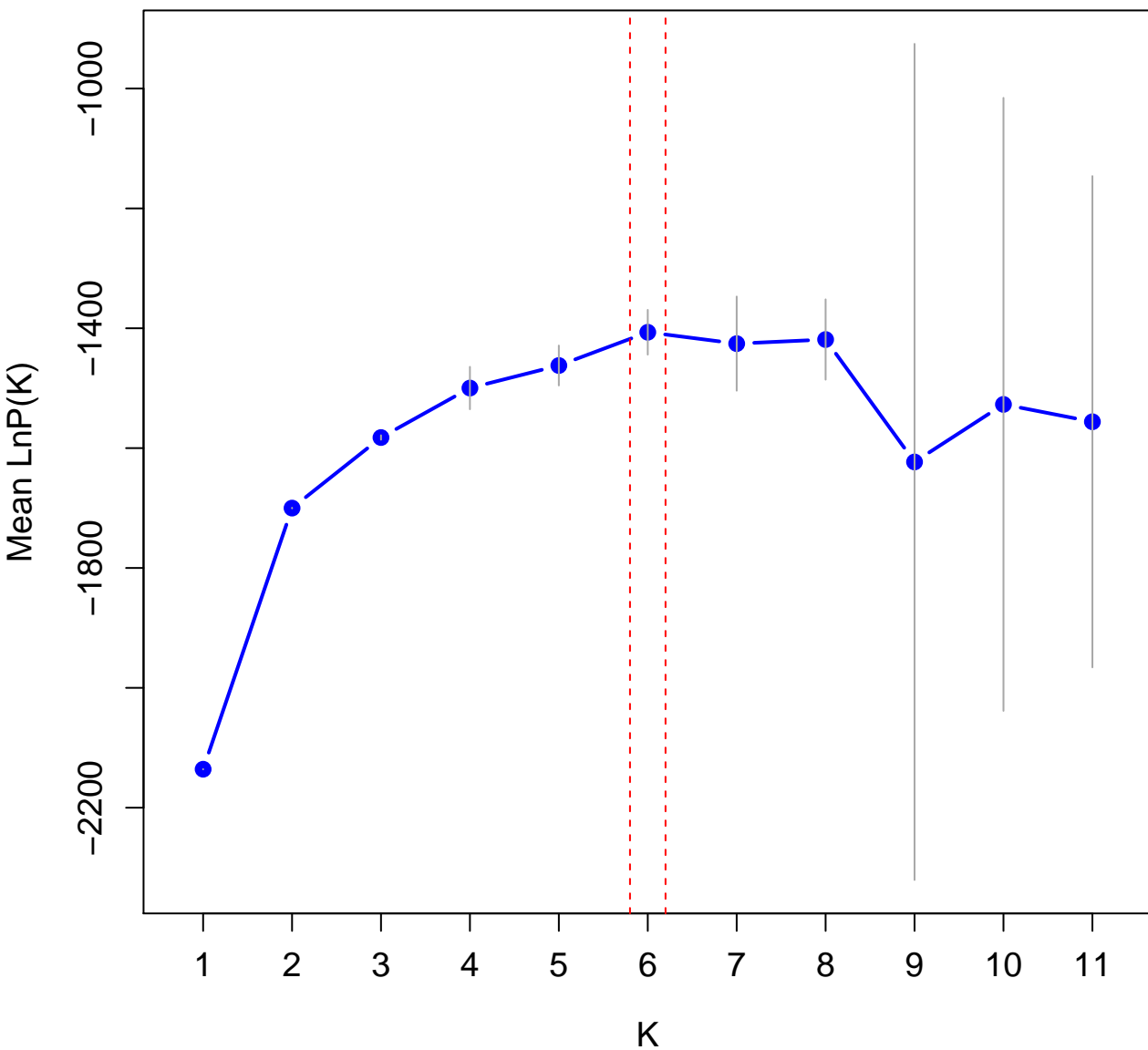

Supplement: Supplementary file 6 — Figure S1. Results of STRUCTURE analysis. Mean estimated log‐normal (Ln) probability of the data in relation to the simulated number of clusters K. Vertical bars indicate standard deviation among ten replicates. [file ECE3-15-e71711-s003.pdf]
